# Examining the association between depersonalisation traits and the bodily self in waking and dreaming

**DOI:** 10.1038/s41598-024-56119-w

**Published:** 2024-03-13

**Authors:** Matt P. D. Gwyther, Bigna Lenggenhager, Jennifer M. Windt, Jane E. Aspell, Anna Ciaunica

**Affiliations:** 1 School of Psychology and Sport Science, Anglia Ruskin University, Cambridge, UK; 2 Department of Psychology, University of Zurich, Zurich, Switzerland; 3 Department of Psychology, University of Konstanz, Konstanz, Germany; 4 Department of Philosophy, Monash University, Melbourne, Australia; 5 Monash Centre for Consciousness and Contemplative Studies, Melbourne, Australia; 6 Centre for Philosophy of Science, University of Lisbon, Campo Grande, 1749-016 Lisbon, Portugal; 7 Institute of Cognitive Neuroscience, University College London, London, WC1N 3AR UK

**Keywords:** Human behaviour, Consciousness

## Abstract

Depersonalisation (DP) is characterized by fundamental alterations to the sense of self that include feelings of detachment and estrangement from one’s body. We conducted an online study in healthy participants (n = 514) with DP traits to investigate and quantify the subjective experience of body and self during waking and dreaming, as the vast majority of previous studies focussed on waking experience only. Investigating dreams in people experiencing DP symptoms may help us understand whether the dream state is a ‘spared space’ where people can temporarily ‘retrieve’ their sense of self and sense of bodily presence. We found that higher DP traits—i.e. higher scores on the Cambridge Depersonalisation Scale (CDS)—were associated with more frequent dream experiences from an outside observer perspective (r = 0.28) and more frequent dream experiences of distinct bodily sensations (r = 0.23). We also found that people with higher CDS scores had more frequent dream experiences of altered bodily perception (r = 0.24), more frequent nightmares (r = 0.33) and higher dream recall (r = 0.17). CDS scores were negatively correlated with body boundary scores (r = − 0.31) in waking states and there was a negative association between CDS scores and the degree of trust in interoceptive signals (r = − 0.52). Our study elucidates the complex phenomenology of DP in relation to bodily selfhood during waking and dreaming and suggests avenues for potential therapeutic interventions in people with chronic depersonalisation (depersonalisation -derealisation disorder).

## Introduction

Transient depersonalisation (DP) experiences, which include fundamental alterations in the experience of one’s bodily self^1–3^, are relatively common in the general population, with a lifetime prevalence rate of 26–74%^4^. People who experience DP describe feeling detached or estranged from their body and self, and sometimes also their surroundings (derealisation)^5–8^. People with persistent depersonalisation experiences are diagnosed with Depersonalisation disorder (DPD), a disturbing psychiatric condition estimated to be present in 1–2% of the population^9^, although it is thought to be under-diagnosed^10^. The study reported here investigated the non-clinical experience of depersonalisation symptoms and their association with aspects of bodily selfhood during waking and dreaming.

Scientists and philosophers distinguish different levels or aspects of self, and have generated a plethora of terms and definitions (see^11^ for a review). ‘Higher’ levels of self include the narrative self and autobiographical self which require language and memory and the experience of a temporally extended existence. These are sometimes contrasted with a ‘lower’ bodily self: the experience of having/being a body. The most basic level of self—the minimal self—has been defined as the experience of being the subject of conscious experience (e.g.,^12^). Another proposal is that minimal phenomenal selfhood involves body ownership, self-identification, and self-location^13^. Drawing on Merleau-Ponty^14^ and William James^15^, many scientists and philosophers now argue that the basic foundation of self, which grounds all layers of self, is anchored in the body^16,17^. Supporting this, there is an emerging scientific consensus that the brain’s representation and integration of multisensory bodily signals plays a central role in structuring the minimal bodily self and in the sense of presence or 'realness' e.g.,^17–19^. Modern scientific attempts to understand the self have thus focused on bodily self-consciousness, i.e., the pre-reflexive experience of being, inhabiting and controlling a body and experiencing the world with the body as the origin of the first person perspective^20^.

Disturbances in bodily self-consciousness are a key feature of DP, with an experienced ‘split’ between the self and the body being one of the most frequently reported symptoms^1^. Sierra^6^ lists four prominent types of anomalous body experiences in DP: (1) loss of body ownership (2) loss of agency (3) disembodiment feelings and (4) somatosensory distortions. The severity of DP symptoms has been related to activation of a number of multisensory brain regions, including parietal cortex^21–23^. The anomalous bodily experiences in DP may therefore be related to abnormal activation in the posterior parietal cortex, an area important for the experience of a stable bodily self^24^. In the current study, building on work suggesting a link between DP and weaker boundaries between self, world and others^3^, we aim to further investigate altered bodily self-consciousness (BSC) in DP, and how this may vary between waking and dream states.

The majority of previous research on DP has focused on atypical BSC during wakefulness. However, little is known about dream experiences in people with DP. This is surprising because many of the individuals with DP describe their daily waking experiences as being “dream-like”, and not feeling fully present or real^6,25,26^. The relationship between dream and waking experience in individuals with DP is so far unclear. Early research by Mayer-Gross^27^ suggests that some people living with persistent DP report relief from symptoms during dreaming, while others report intense DP and derealisation experiences in dreams. Investigating dreams in people experiencing DP symptoms may help us better understand whether the dream state is a ‘spared space’ where people can temporarily ‘retrieve’ their sense of self and sense of presence. In the current exploratory online study, we investigated how waking DP traits are related to the experience of body and self in dreams by measuring DP traits using the Cambridge Depersonalisation Scale (CDS) and asking participants to answer questions about their self and body dream experiences.

Humans spend roughly a third of their lives asleep and much of this time is spent dreaming. Dreams are spontaneous mental simulations that involve the experience of a self in a world and have been described as the quintessential virtual reality^28^. Simulation theories of dreaming, which are now widely accepted, describe dreams as ‘self in a world’ experiences^29^. Yet, self-experience in dreams, while highly variable, is associated with profound alterations compared to waking, including dissociative experiences and alterations in cognition, metacognition and bodily experiences^30,31^.

Certain types of less common dream experiences involving the body^31^, such as ‘outside observer’ dreams, dreams with distinct bodily sensations, and changes to bodily perception, have parallels with the types of anomalous bodily experiences found in waking DP. These types of dream experiences are memorable due to their sometimes disturbing and nightmarish quality. Indeed, nightmares occurring during sleep or sleep onset often include disturbing bodily sensations such as paralysis, difficulty moving and vestibular sensations such as falling^32,33^. If there is continuity between disturbing waking DP experiences and dreams, we might expect an increased incidence of nightmares in people with high DP traits.

Finally, differences in the processing of signals from inside the body (interoceptive signals) have also been studied in DP, although results have been mixed^34–36^. Bodily self-consciousness arguably emerges from the integration of interoceptive (internal) and exteroceptive (external) signals see e.g.,^37^, and disruptions in this integration may explain some symptoms of DP^38^. Whether interoception is disrupted in DP, and which aspects of interoception might be affected is not yet certain: previous studies, which showed mixed results, relied on heartbeat detection tasks as a behavioural measure of interoceptive accuracy^35,39^ or brain responses to heartbeat signals^36^, and these do not always reliably correlate with measures of subjective interoceptive awareness^39^. To further explore the relationship between DP and interoception, in this study we used self-report measures (questions relating to noticing and trusting of interoceptive signals) from the Multidimensional Assessment of Interoceptive Awareness (MAIA-2) questionnaire^40^. We predict that (1) people with higher CDS scores will trust signals from their body less while awake, and (2) people with high DP traits will notice bodily signals less while awake. See Table 1 for a summary of all hypotheses for the study.

**Table 1:** Hypotheses.

1a	Participants with higher DP traits (as measured by CDS-total score) will report that they trust internal bodily signals less while awake
1b	Participants with higher DP traits will report that they notice internal bodily signals less while awake
2a	Participants with higher DP traits will report that they have more dream experiences from an outside observer perspective
2b	Participants with higher DP traits will report that they are less aware of the presence (or absence) of their body in dreams
2c	Participants with higher DP traits will report that they have more dream experiences of distinct bodily sensations
2d	Participants with higher DP traits will report that they have more dream experiences of alterations in bodily perception
3	Participants with higher DP traits will report that the boundaries of their body are more permeable while awake
4	Participants with higher DP traits will report that their sense of self is more separate from others while awake
5	Participants with higher DP traits will report more frequent nightmares
6	Participants with higher DP traits will report more frequent dream recall

In summary, this exploratory study aimed to investigate whether DP traits (in a non-clinical sample) are associated with changes to different aspects of bodily self-consciousness during waking and dreaming and whether DP traits are associated with increased nightmare incidence and dream recall.

## Methods

### Participants

Based on data from an online pilot study (n = 9), we estimated that the conversion rate from pre-screening would be between 20 and 25% based on an estimated 50% pass rate of screening criteria and a 50% start rate from those who passed screening. Therefore, the target for the prescreening survey was 400–500 complete responses.

A total of 860 healthy adult participants responded to the survey. We included inclusion/ exclusion criteria to ensure this: “Are you taking antidepressants OR antipsychotics?” and “Are you suffering from a seizure disorder?” If participants replied ‘yes’ to either they were excluded from the sample. 514 participants completed the full survey (aged 18–75 years-old (M = 32.7, SD = 11.84); 113 men, 390 women, 9 participants used ‘Prefer to self-describe’; and 1 ‘Prefer not to say’). Most participants reported their ethnicity as ‘Caucasian European’ (71.4%; South-Asian 5.1%, East-Asian 2.9%, Black African 2.7%, Caucasian American 2.5%; combined other categories 15.4%). The pre-screening phase took place in two steps: (1) in November 2021 via social media and a previous subject pool of participants that took part in previous studies led by one of us (AC) and agreed to be recontacted for follow up studies (n = 605); (2) Prolific Academic between January 7 and 20, 2022 (n = 255).

### Procedure

The study was conducted online and was first approved by the School of Psychology and Sport Science Ethics Panel at Anglia Ruskin University, Cambridge, UK. The total survey took approximately 10–15 min, and it was conducted in accordance with the Helsinki Declaration. All participants provided informed consent after reading a participant information sheet.

### Measures

The online survey was hosted on the software platform Qualtrics and contained a participant information sheet, a consent form, demographic questions (age, gender, ethnicity) and several measures of interest presented in the same order for each participant (see below). The participants could complete the survey at any time of day on a computer or mobile device.

#### Cambridge Depersonalisation Scale

The Cambridge Depersonalisation Scale (CDS-29)^41^ is a 29-item self-report measure developed to determine the severity of depersonalisation experiences. The CDS asks participants to rate both the frequency and intensity of depersonalisation experiences over the past 6 months. Frequency is measured on the following scale “never”, “rarely”, “often”, “very often”, “all the time” and Duration on the scale “for a few seconds”, “for a few minutes”, “for a few hours”, “for about a day”, “more than a day”, “all the time”. The total score is calculated by summing all items (0–290 points). The scale has good psychometric properties with Cronbach’s alpha between 0.89 and 0.94 in previous language versions^41–45^. A calculation of the internal reliability of our CDS-29 scores revealed a Cronbach’s alpha of 0.97.

Sierra et al.^45^ extracted four subscales from the CDS-29: anomalous Body Experience, Emotional Numbing, Anomalous Subjective Recall, and Alienation from Surroundings. In our sample, the subscales correlated (between 0.66 and 0.88) with each other more strongly than in Sierra et al. (between 0.23 and 0.34), which may result from different sampling methodologies (online vs. in-person). In this study, we report both the CDS-29 total score and the four CDS subscales.

### Dream-related items

#### Short bodily experience in dreams questionnaire

Building upon a previously published Bodily Experience in Dreams Scale^46^, we developed four single-item self-report measures to assess participants’ reported differences in bodily experiences during dreams. The measures were all presented on a 0–100 visual analogue scale where 0 is never and 100 all the time (see Table 2).

**Table 2:** Dream-related items.

Title	Item	Scale
Bodily experience in dreams (1): outside observer	“I experience dreams from an outside (observer) perspective, as if I were not present in the dream”	Visual analogue 0–100
Bodily experience in dreams (2): body presence/absence	“In my dreams I am aware of the presence (or absence) of my body”,	Visual analogue 0–100
Bodily experience in dreams (3): distinct bodily sensations	“In my dreams I have distinct bodily sensations (like pain, touch)”	Visual analogue 0–100
Bodily experience in dreams (4): body perceived differently	“The way I perceive my body in dreams is different (e.g., in terms of shape, functions) from the way I perceive my body in waking state”	Visual analogue 0–100
Nightmare frequency	“On average how often have you experienced nightmares over the past 6 months?”	7-point Likert scale
Dream recall	“Considering the last month, how often did you recall your dreams in the morning?”	7-point Likert scale

#### Nightmare recall frequency

We adapted a single-item self-report measure to assess how often participants recalled nightmares over the past 6 months^47^ (Table 1). The measure was presented on a 7-point Likert scale (1 “Never”, 2 “less than once a month”, 3 “about once a month”, 4 “twice or three times a month”, 5 “about once a week”, 6 “several times a week”, 7 “almost every night”).

#### Dream recall frequency

We used a single-item self-report measure to assess how often participants recalled their dreams over the past month^48^ (Table 1). The measure was presented on a 7-point Likert scale (1 “Never”, 2 “less than once a month”, 3 “about once a month”, 4 “twice or three times a month”, 5 “about once a week”, 6 “several times a week”, 7 “almost every morning”).

### Waking-related items

#### Visual analogue scale assessing perceived body boundaries

**Figure 1:**
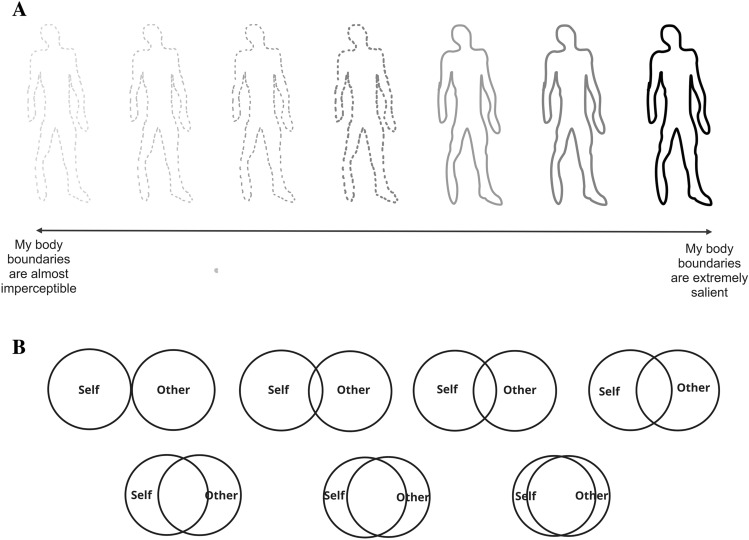
(**A**) Visual analogue scale assessing perceived body boundaries. Dambrun’s^49^ single-measure self-reported perceived body boundaries scale is used to assess participants’ current perceived body state. It depicts seven bodies in a row, the furthest left has almost imperceptible boundaries and the furthest right has extremely salient boundaries (**A**) Participants were presented with the measure on a 0–100 visual analogue scale and asked to drag a slider to the position best representing their current body state. (**B**) The inclusion of other in the self (IOS) scale^50^ is a single-item self-reported scale used to assess how close participants feel to other people. Participants were presented with seven pairs of circles that range from barely touching to almost completely overlapping and were asked ‘Which picture best describes your relationship with others (in general)’ (**B**).

Dambrun’s^49^ single-measure self-reported perceived body boundaries scale is used to assess participants’ current perceived body state. It depicts seven bodies in a row, the furthest left has almost imperceptible boundaries and the furthest right has extremely salient boundaries (see Fig. 1A) Participants were presented with the measure on a 0–100 visual analogue scale and asked to drag a slider to the position best representing their current body state.

#### Inclusion of other in the self (IOS) Scale

The Inclusion of Other in the Self (IOS) Scale^50^ is a single-item self-reported scale used to assess how close participants feel to other people. Participants were presented with seven pairs of circles that range from barely touching to almost completely overlapping and were asked ‘Which picture best describes your relationship with others (in general)’ (see Fig. 1B).

#### Multidimensional assessment of interoceptive awareness (Version 2)

The Multidimensional Assessment of Interoceptive Awareness (MAIA-2)^40^ is a 32-item self-report multidimensional measure of interoceptive awareness made up of eight subscales. For the purposes of our study, we selected the Noticing and Trusting sub-scales. The Noticing subscale comprises 4-items and assesses subjective awareness of bodily sensations (e.g., “I notice when I am uncomfortable in my body”). The Trusting subscale is comprised of 3-items and assesses the degree to which a person considers their body as ‘safe’ (e.g., “I am at home in my body.”) Responses for the two selected MAIA subscales were given on a 6-point scale from “never (0)” to “always (5)”. Scores were calculated as the mean of all items within a given subscale. Cronbach alphas for Noticing (0.75) and Trusting (0.86) indicated good internal consistency for our use of these scales, consistent with Brown et al.^51^ (Noticing = 0.76 and Trusting = 0.92) and beyond those reported in the original MAIA-2 paper (^40^; N = 0.64 , T = 0.83).

### Statistical analysis plan

As our primary aim was to understand the relationship between waking depersonalisation traits and a number of facets of waking and dreaming bodily experience, we planned to measure the association between CDS-29 total scores and all other study variables. We also planned to explore the relationship between CDS subscale scores and all other study variables as an exploratory piece of analysis outside of our set of predictions. Due to non-normality of CDS-29 score data, Spearman’s rank correlations were conducted to explore the relationship between CDS-29 total scores and subscales and all the other study variables (see Table 1). Due to the number of comparisons, we controlled for false discovery rate (FDR) using the Benjamini and Hochberg^52^ procedure which adjusts p-values of multiple tests so that the proportion of false positives is limited. All correlations are significant at p < 0.001.

## Results

### Descriptives

For our sample of 514 participants (113 men, 390 women), the mean total CDS-29 score in our sample was 54.8 (SD = 47.3), with median at 42 and mode at 30. Range was between 0 and 255. The CDS-29 total score distribution was strongly positively (right-sided) skewed (skewness = 1.45) and slightly leptokurtic (Kurtosis = 2.08) with the majority of participants recording relatively low scores.

Descriptive statistics for the CDS-29 subscales and other study variables are reported in Tables 3 and 4.

**Table 3:** Descriptive statistics for CDS-29 subscales.

CDS-29 subscale	N	Mean	Standard deviation	Median
Anomalous body experience (ABE)	514	13.2	15.4	8
Emotional numbing (EN)	514	11.2	11.9	7
Anomalous subjective recall (ASR)	514	12	9.2	10
Alienation from surroundings (AFS)	514	11	8.2	10

**Table 4:** Descriptive statistics for other study variables.

Subscale	N	Mean	Standard deviation	Median
BID (1) Outside observer	510	24	24.7	19
BID (2) Body presence/absence	510	45.8	34.2	50
BID (3) Distinct bodily sensations	509	34	32.1	21
BID (4) Body perceived differently	508	35.3	32.6	25
Nightmare frequency	514	2.8	1.5	2
Dream recall	513	4.9	1.7	5
Perceived body boundaries	514	70.5	24.3	75
Inclusion of other in the self (IOS)	513	3.4	1.6	3
MAIA(2)—noticing	513	3.3	1	3.5
MAIA(2)—trusting	513	3.4	1.2	3.7

Figures are reported at 1dp.

### Correlation analysis

**Figure 2:**
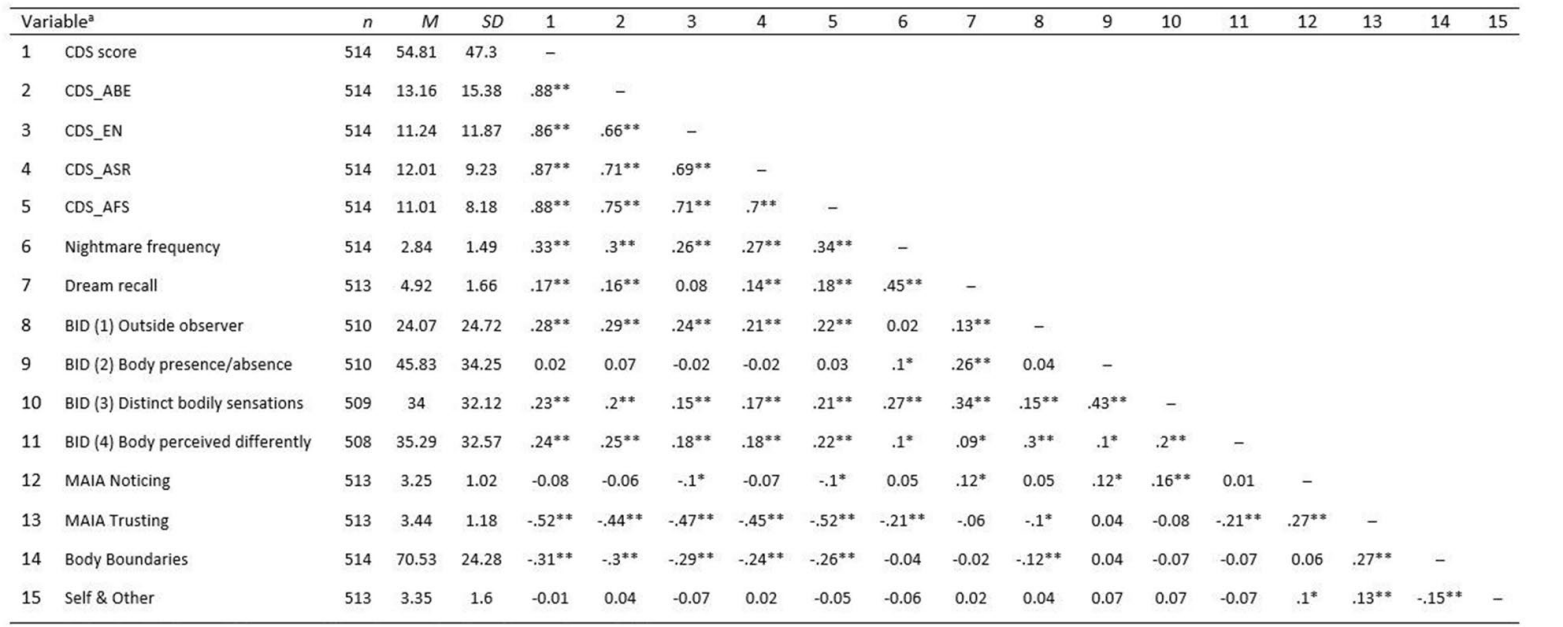
Descriptive statistics and Spearman’s rank correlations for study variables. *p-FDR < 0.05; **p-FDR < 0.01. (1) CDS Score = ‘Cambridge Depersonalisation Scale’ score, (2) CDS_ABE = ‘anomalous body experience’, (3) CDS_EN = ‘emotional numbing’, (4) CDS_ASR = ‘anomalous subjective recall’, (5) CDS_AFS = ‘alienation from surroundings’, (6) Nightmare frequency, (7) Dream recall, (8) Body in dreams (1) = experiencing dreams from an outside (observer) perspective, (9) Body in dreams (2) = aware of the presence (or absence) of body, (10) Body in dreams (3) = distinct bodily sensations (like pain, touch), (11) Body in dreams (4) = perceive body in dreams differently (e.g. in terms of shape, functions) from waking state (12) MIAI Noticing = ‘Multidimensional Assessment of Interoceptive Awareness’, Noticing (13) MIAI Trusting = Trusting, (14) Body Boundaries = Visual analogue scale assessing perceived body boundaries, (15) Self and Other scale.

The full set of correlations, including exploratory CDS-29 subscale correlations, are reported in Fig. 2 along with corrected p-values. These results are shown alongside our hypotheses in Table 5. We ran additional analyses on two subsets of the sample and report these in our Supplementary information in Supplementary Table S1. The two data subsets include: (1) removal of all 0 total CDS scores (n = 12) for a sample of (n = 502) and (2) removal of total CDS scores under 50 (n = 291) for a sample of (n = 223). This additional analysis was conducted in order to assess the relationships between measures for participants with at least mild DP symptoms as measured by total CDS score.

**Table 5:** Study hypotheses alongside results.

Hypotheses		Supported by results	Correlation (Spearman’s Rho)
1a	Participants with higher DP traits (as measured by CDS-total score) will report that they trust internal bodily signals less while awake	Yes	r = − 0.52
1b	Participants with higher DP traits will report that they notice internal bodily signals less while awake	No	r = − 0.08
2a	Participants with higher DP traits will report that they have more dream experiences from an outside observer perspective	Yes	r = 0.28
2b	Participants with higher DP traits will report that they are less aware of the presence (or absence) of their body in dreams	No	r = 0.02
2c	Participants with higher DP traits will report that they have more dream experiences of distinct bodily sensations	Yes	r = 0.23
2d	Participants with higher DP traits will report that they have more dream experiences of alterations in bodily perception	Yes	r = 0.24
3	Participants with higher DP traits will report that the boundaries of their body are more permeable while awake	Yes	r = − 0.31
4	Participants with higher DP traits will report that their sense of self is more separate from others while awake	No	r = − 0.01
5	Participants with higher DP traits will report more frequent nightmares	Yes	r = 0.33
6	Participants with higher DP traits will report more frequent dream recall	Yes	r = 0.17

(i) Dreaming

**Figure 3:**
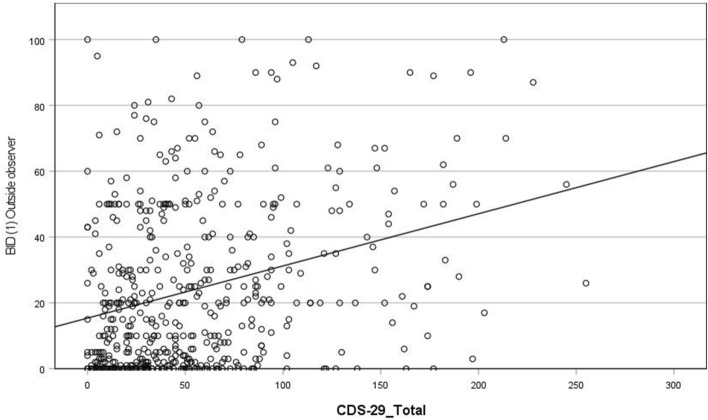
Scatterplot of correlation between CDS-29_Total score and Bodily Experience in Dreams (1) Outside observer.

**Figure 4:**
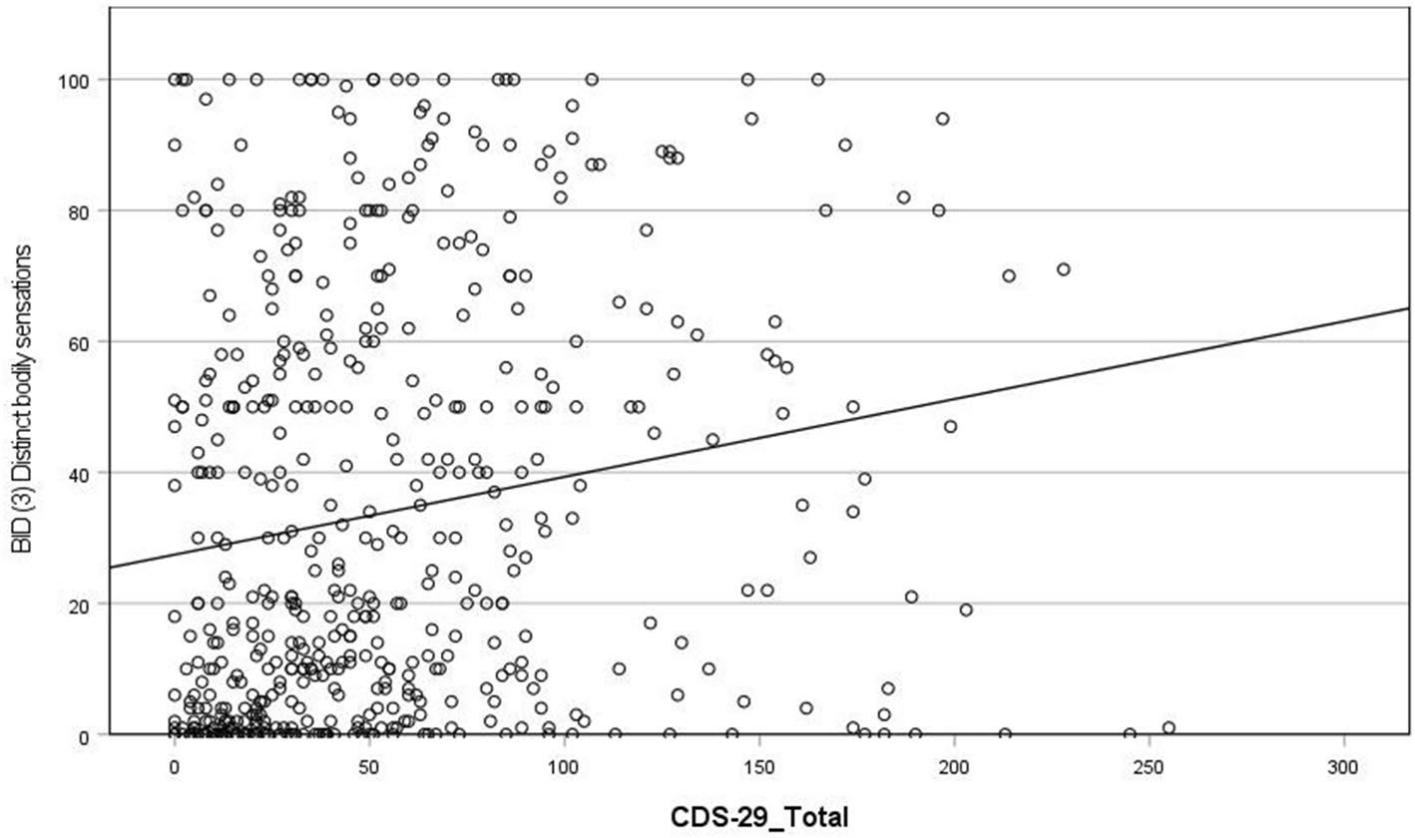
Scatterplot of correlation between CDS-29_Total score and Bodily Experience in Dreams (3) Distinct bodily sensations.

**Figure 5:**
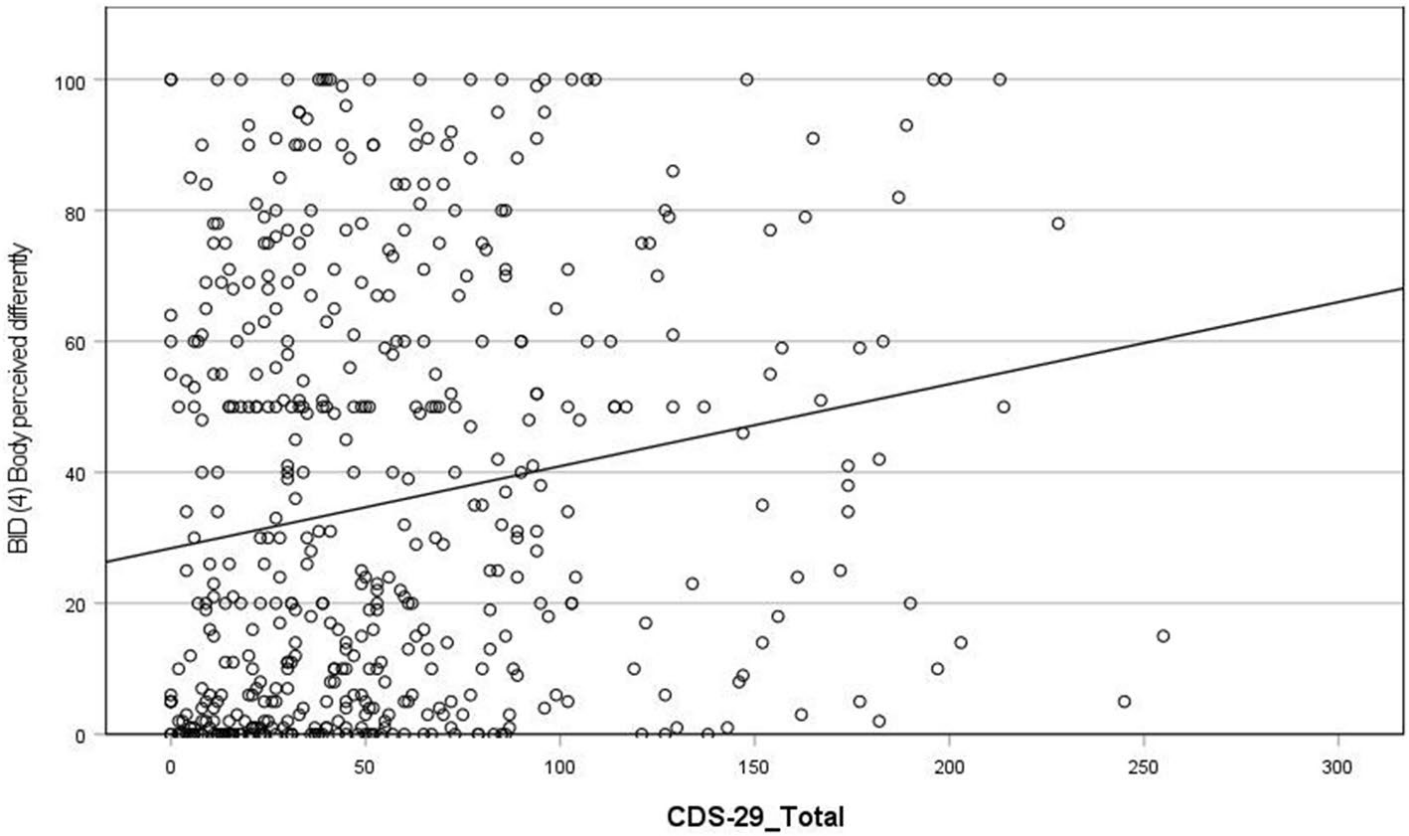
Scatterplot of correlation between CDS-29_Total score and Bodily Experience in Dreams (4) dream experiences of alterations in bodily perception.

Results indicate that higher CDS total scores are associated with more frequent dream experiences from an outside observer perspective (r = 0.28; see Fig. 3). In addition, higher CDS scores are associated with more frequent dream experiences of both (i) distinct bodily sensations (r = 0.23; see Fig. 4) and (ii) alterations in bodily perception (r = 0.2; see Fig. 5). There was no association between CDS scores and awareness of the presence or absence of the body during dreaming. Note: when we removed participants from the sample who scored less than 50 total CDS score, the relationship between total CDS scores and dream experiences of distinct bodily sensations was no longer significant (see Supplementary Table S1 and “Discussion”).

(ii) Nightmare frequency and dream recall

Higher CDS scores were associated with more frequent nightmares (r = 0.33) and more frequent dream recall (r = 0.17). Note: when we removed participants from the sample who scored less than 50 total CDS score, the relationship between total CDS scores and nightmare frequency was significant but weaker and the relationship between total CDS scores and nightmare frequency was no longer significant (see Supplementary Table S1 and “Discussion”).

(iii) Waking experiences

**Figure 6:**
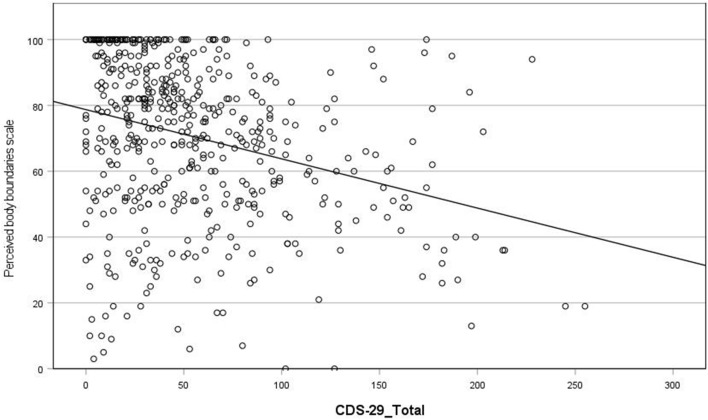
Scatterplot of correlation between CDS-29_Total score and Perceived body boundaries scale.

**Figure 7:**
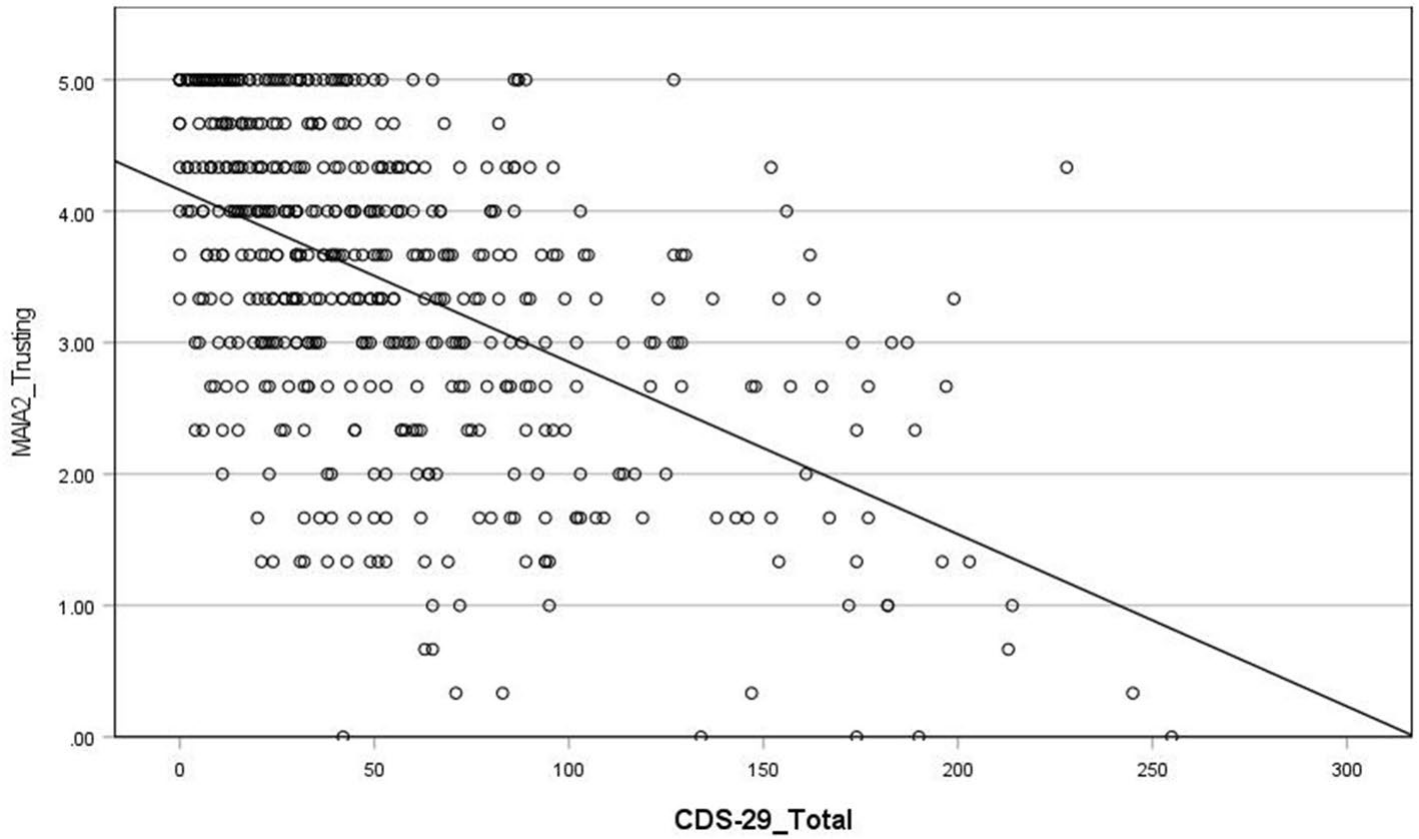
Scatterplot of correlation between CDS-29_Total score and MAIA2_Trusting subscale.

CDS scores were significantly negatively correlated with the perceived body boundaries score (r = − 0.31) (Fig. 6), such that higher CDS scores are associated with more permeable body boundaries during waking. There was no correlation between CDS scores and scores on the Inclusion of Other in the Self (IOS) Scale. Results show a significant negative association between CDS scores and the interoception measure MAIA-2 subscale ‘Trusting’ (r = − 0.52) (Fig. 7). There was no significant association between CDS scores and the MAIA-2 subscale ‘Noticing’. Note: when we removed participants from the sample who scored less than 50 total CDS score, the relationship between total CDS scores and MAIA-2 Trusting was significant but weaker (see Supplementary Table S1 and “Discussion”).

## Discussion

This study examined the relationship between waking DP traits in healthy participants and measures of the experience of the bodily self during both waking and dreaming. The study yields several results, which are discussed in detail in the following. A consensus score of ≥ 0.4 or ≤ − 0.4 is used to determine whether a correlation is described as moderate or weak. Scores of ≥ 0.5 or ≤ − 0.5 are referred to as strong correlations.

### Dreaming and DP

We investigated the relationship between waking DP symptoms and bodily experiences during dreaming. We found that higher total CDS scores are weakly associated with (i) more frequent dream experiences from an outside observer perspective, (ii) more frequent dream experiences of distinct bodily sensations (like pain and touch) and (iii) more frequent dream experiences of alterations in bodily perception (e.g., in terms of shape and functions). There was no association between CDS scores and awareness of the presence or absence of the body during dreaming. Finally, we found that higher CDS scores are moderately associated with more frequent nightmares and weakly associated with more frequent dream recall. Additional analysis including only participants who scored 50 or above on total CDS score changed the significance and effect size for some study variables as outlined in our results section and Supplementary Table S1. In this study, we were primarily interested in the full range of DP traits in this non-clinical sample, however we hope the inclusion of analyses for people with at least mild DP traits may inspire future studies with clinical samples.

In what follows we discuss the implications of these findings in turn.

#### Bodily experience during dreaming

Our findings suggest some degree of continuity between waking DP symptoms and specific aspects of bodily and self experience in dreams. We found a weak correlation between CDS score and item 1 of our shortened BED questionnaire: "I experience dreams from an outside (observer) perspective, as if I were not present in the dream." This finding suggests that waking DP, in particular in relation to the anomalous bodily experience subscale (where the correlation was highest) is associated with differences in the experience of presence during dreaming. However, we found no significant correlation between CDS scores and responses to the statement: “In my dreams I am aware of the presence (or absence) of my body”. One potential interpretation here is that the felt presence or the absence of the body may be related to the experience of an acting body in the world. During sleep, our bodies are mostly passive, hence the feelings of presence or absence may be diminished. It would be interesting to examine this aspect in relation to deep meditative experiences, where the body is in a still position for a longer time, to see whether the feeling of the presence or absence of the body also occurs (Becattini et al. in prep.)

Our findings therefore suggest a mixed picture on whether dreaming may be considered a ‘spared space’ allowing people with high DP traits to ‘regain’ a sense of presence. Considering both findings together, the association we found between CDS scores and experiences of dreams from an outside observer perspective, may suggest that differences in self experience during dreaming may be more rooted in changes to spatiotemporal self-location rather than embodiment. Viewed through Windt’s^53^ immersive spatiotemporal hallucination model of dreaming (ISTH), the findings may indicate that DP traits are associated with increased incidence of experiences similar to neurological out of body experiences (OBEs;^54^) and full body illusions induced using virtual reality^55,56^). Further work is needed to examine the phenomenal character of these ‘outside observer experiences’ dreams in DP.

Another possible interpretation can be put forward in relation to the recent proposal linking DP experiences with the phenomenon of hyper-reflexivity or ‘over-thinking’ about oneself^2,82,83^. Indeed, feelings of being ‘stuck’ in one’s head and outside one’s body are very common in DP^25,94,95^. If these hypotheses are correct, then depersonalisation symptoms, although typically couched as “losing” one’s sense of self, may be linked, on the contrary, to an inability to attenuate self-related inputs and hence to ‘forget’ the self in the background^2^. Alterations in the ability to attenuate self-related information in order to optimally perceive, engage and act in the world may further lead to increased reflexivity or ‘hyper-reflexivity’^26,82,83^. For example, over-attending to one’s leg movements while running may prompt people to detach themselves from the action, and see themselves from ‘above’, from a third-person observer perspective. This bias towards self-related over-thinking and hyper-reflexivity, offsets diminished body-related processing. This hypothesis is consistent with subjective reports outlining feelings of being simultaneously trapped in one’s head (mind) and outside one’s body (disembodiment)^2,26^. During sleep however, the higher-order self-related processing is diminished, and hence this may explain the prevalence of body-related processing in DP dream states. Lower active engagements with the external world via the ‘outer’ senses such as vision during sleep may lead to heightened ‘inner’ processing, with one’s body at the centre of one’s experiences. Again, based on the interpretation proposed by Ciaunica and colleagues^2^, one may predict that the more suppressed internal bodily signals are during wakefulness in DP (i.e. detachment from one’s visceral sensations), the higher the presence of body-related signals during dreams in DP, as suggested by our findings, discussed below.

Regarding bodily experience during dreaming, we found that those with high CDS scores were more likely to report dream experiences of both distinct bodily sensations (like pain and touch) and changes in bodily perception (in terms of shape and function) which are usually rare in dreams^57^. Our finding thus seems to suggest that there is heightened processing and/or awareness of bodily signals during dreams in high DP individuals. These findings also indicate continuity (between waking and dreaming) of specific aspects of bodily experience that are present in waking DP, such as changes to bodily function^3^. Further work is needed to understand the precise nature of these experiences by acquiring detailed qualitative dream reports. Our findings lend credence to the idea that dreams are heterogeneous and variable phenomena during which the representation of self varies considerably^58,59^. Our study adds to a small but growing number of studies that investigate dreams in ‘non-typical’ states, e.g., amputees, deaf, blind, paraplegic, schizophrenia^60–64^. The body of research examining dream experiences across varying physical and psychological states provides intriguing insights into the validity of the continuity hypothesis (Table 6).

**Table 6:** Dreams in non-typical states.

Authors	Key findings	Evidence for continuity hypothesis
Bekrater-Bodmann et al. (2015)^60^	Post-amputation pain, specifically phantom limb pain (PLP) and residual limb pain (RLP) were positively associated with recalling an impaired body representation in dreams. However, subjects had a mix of experiences with some not recalling their body in dreams, some with their bodies always intact and fewer with their bodies always impaired	Mixed
Saurat et al. (2011)^61^	Despite the absence of walking experiences in waking life, individuals with paraplegia report frequent and vivid walking dreams, which could be seen as a reflection of their desire or conceptual understanding of walking	Mixed
Voss et al. (2011)^62^	Individuals with sensory impairments, such as congenital deafness or paraplegia, report dream content that is strikingly similar to those without such impairments	No
Lusignan et al. (2009)^63^	Individuals with schizophrenia experience a higher incidence of nightmares and an altered perception of dream bizarreness, which may mirror their waking emotional experiences and cognitive peculiarities	Yes
Hurovitz et al. (1999)^64^	Visual content in dreams seems to be contingent on the dreamers' lifetime visual experiences with congenital and early childhood blindness, total blindness and high percentage of total life spent in blindness negatively correlating with visual references during dreaming	Yes

Table represents a non-exhaustive selection of papers on dreams in non-typical states, covering a range of conditions.

Collectively, these studies suggest that while dream content is broadly continuous with waking life, it can also incorporate elements beyond the dreamers' lived experiences, reflecting a complex interplay between waking reality, cognitive schemas, and individual psychopathology. We extend this idea to non-typical states in a non-clinical population, indicating further aspects of continuity of bodily experience and self representation between wakefulness and dreaming, while indicating a complex interplay of self-related processing and individual differences.

Overall, our findings suggest some support for the continuity hypothesis, i.e. the idea that dreams are continuous with waking experiences, with the suggestion that aspects of waking DP experience, such as experiencing the body from the third person perspective and changes to bodily perception, may also be present during dreaming. It is also consistent however with the idea that hyper-reflexivity being put on hold during sleep, deeper, bodily levels of self-related processing can finally take center stage.

However, given that the correlations between DP traits and the extent of ‘DP-like’ experiences during dreams are weak to moderate, future work is needed to investigate this.

#### Nightmares, dream recall and DP

Consistent with previous empirical findings linking DP with increased occurrence of nightmares^65^ our study revealed that participants with higher DP traits reported more frequent nightmares and more frequent dream recall. Indeed, given the often disturbing waking phenomenology of anomalous bodily experiences such as disembodiment in DP^6^ and accompanying threats to the integrity of felt selfhood, one would expect dreams to simulate aspects of this experience and present as more frequent nightmares. This is made more likely by the fact that nightmares often involve distinct and disturbing feelings of paralysis and difficulties moving, as well as vestibular sensations^32,33^ and these have overlaps with some of the disturbing DP phenomenology. Further work is needed to investigate nightmare phenomenology in DP and to establish its links with similar aspects of waking bodily experience.

Recent studies have shown a correlation between COVID-19-related nightmares and dissociative experiences^66^ as well as a positive correlation between dissociative experiences and nightmares in participants without mental diagnoses^67^. Furthermore, Levin and Nielsen^68^ outlined how psychiatric symptoms in waking life, particularly in relation to PTSD, can affect the prevalence and intensity of nightmares. Agargun et al.^69^ also found that nightmares were common for children experiencing trauma and dissociation, showing slight evidence for dissociation being useful to lessen the burden of trauma in nightmares. Taken together, this suggests that the link between depersonalisation and nightmares found in our study is likely part of a more complex interaction between trauma, dissociation, and nightmares.

### Waking and DP

#### Body boundaries

In the waking component of our study, we found a significant, moderate negative association between CDS score and perceived body boundaries, further indicating that high DP traits are associated with the experience of less salient body boundaries, or put another way, weaker boundaries between self and the world^3^. Interestingly, the measure we used for body boundaries was originally used in a study indicating that a body scan meditation can reduce the salience of perceived body boundaries and promote happiness^49^, which is in line with evidence suggesting the ability of long-term meditators to flexibly modulate bodily self-awareness^70,71^. The relationship between meditation and depersonalisation experiences is complex^26,72^. Disruptions to the bodily self can occur in both pathological and desired "enlightened" experiential states^73^, and there are likely to be a multitude of phenomenological differences between conscious states described in such terms^74^. Much of the meditation literature describes the positive impact of loss of self boundaries^49,75^, but the risks associated with this type of "self-loss" experience have also been flagged^72^. On the one hand, meditation may help to improve aspects of interoceptive awareness and stability in the sense of self and body, potentially reducing depersonalisation symptoms. On the other hand, the heightened focus on internal sensations and lack of movement during static meditation may exacerbate depersonalisation experiences, particularly for individuals with a history of trauma or preexisting disruptions in bodily self-awareness^26^.

Intriguingly, we report a significant moderate positive correlation between the trusting measure of interoception and perceived body boundaries (Table 5), indicating that an increased trust in bodily signals may be linked to a stronger sense of one's body boundaries. This may be linked to previous findings^76,77^ that people with higher interoceptive sensitivity are less prone to body illusions. Our findings suggest that unpacking the relationship between body boundaries, trust in interoceptive signals and DP may be fruitful avenues for future research.

### Interoception and DP

Turning to our findings relating to interoception, we found a moderate negative association between CDS scores and the degree of *trust* in interoceptive signals. However, no significant correlation between CDS scores and *noticing* of interoceptive signals in wake states was found. This lack of difference in noticing of interoceptive signals may shed light on inconsistent findings on interoceptive accuracy and its relation to depersonalisation experiences^35,39^. Although in a non-clinical sample, we speculate that our findings may support the suggestion that previous inconsistent findings when measuring interoceptive awareness may be due to difficulties that DP creates in sustaining attention to interoceptive signals rather than the noticing of signals as such^39^. Interestingly, although the noticing of cardiac signals may be similar for people with high and low DP traits, our results suggest that those signals may be deemed less trustworthy for people with high DP. These results are in line with self-reports of people with DP as living inside one’s head and outside one’s body^26,78^.

The influential Predictive Processing framework^78^ has been recently used by several authors to explain abnormal interoceptive processing in relation to the phenomenology of the loss of "mineness" often experienced in DP^17,79,80^. For example, Seth, Suzuki & Critchley^80^ proposed that DP is a disorder of presence resulting from imprecise interoceptive predictive signals. More recently, Ciaunica and colleagues^3^ proposed a novel PP interpretation of DP suggesting that DP experiences, although typically couched as “losing” one’s sense of self, may be linked, on the contrary, to an inability to sensory attenuate self-related inputs and hence to ‘forget’ the self in the background while acting in the world^3,26^. Alterations in the ability to attenuate self-related information in order to optimally perceive, engage and act in the world may further lead to increased reflexivity or ‘hyper-reflexivity’^26,81–83^. Importantly, during sleep states, our reflective capacities are reduced, and the bodily self is processed in the background, for survival purposes (e.g., we don’t stop breathing as soon as we lose consciousness of our breathing).

One of the key points highlighted by Ciaunica and colleagues^3^ is that living systems such as human bodies need to engage actively with the environment for survival, hence self-awareness necessarily needs to include dynamic processing in its very core. From an evolutionary perspective, humans need to actively engage with the environment to nourish themselves, hence the metabolic systems during sleep may be diminished in order to save energy for when ‘it makes sense’ to activate those systems (i.e. when one is awake and able to seek for food).

While previous predictive processing approaches outlined the interoceptive^84,85^, or affective facets of selfhood^79,86^, the new model takes into account the idea that the human body cannot achieve self-regulation of internal (interoceptive) states without maintaining and engaging in active exchanges with its ‘external’ proximal environment^2,87,88^. Indeed, our bodily self is not a static and closed entity, but rather a dynamic and open system, literally constituted in relation to a proximal environment^2,89^. Hence, somatosensory attenuation becomes a key part of the story of understanding how the self merges as differentiated and yet related to its surroundings.

If this is so, then our results in this study might suggest that people with high DP scores experience a split between their ability to subjectively trust their body to act in the world out there, while paradoxically they are able to objectively use their bodies to function ‘mechanically’ or “on automatic pilot’ in their daily life^25^. Our findings suggest that there may be no significant difference in noticing bodily signals between people with high and low levels of DP, which seems to suggest that they do not ‘lose’ track of their bodies, rather they don’t seem to trust their bodies. These findings are in line with the phenomenological approaches proposing that DP may be related with people feeling their bodies more like objects of an experience rather than subjects of an experience^26^.

Future work needs to disentangle the complex multifaceted aspects of the relationship between various aspects of interoception and DP, specifically in relation to interoceptive awareness and bodily movements. Specifically, if future studies that employ multiple measures of interoception show that deficits in interoceptive processing are reliably associated with DP, then potential therapeutic approaches for DP may include interventions that aim to build trust in bodily signals^34^.

### Limitations and outlook

Due to the self-selection of the participants we cannot exclude that the sample is biased towards people with an interest in dreams. However, this is a common methodological challenge in dream research across lab and online studies. An additional limitation of this study is its reliance on self-report measures. Such self-report measures can be subject to biases and, of particular relevance in relation to dreams, failures in memory. We also used some bespoke measures (questions) for bodily experience in dreams, which although based on previous empirical work, have not been validated. Furthermore, as the study is correlational, we cannot be sure that DP traits cause the differences in dreams observed.

Another important limitation of this study is that we examined participants' general reports of bodily experiences in dreams, as well as a ‘high-level’ measure of interoception (interoceptive sensibility), through responses to an online questionnaire. In order to further investigate the relationships between DP, dreams and interoception we encourage further research using ‘low-level’, objective measures^96^ such as heartbeat perception tasks or heartbeat evoked potentials. It is also a limitation that the measures used to ascertain bodily experiences in dreams may not accurately reflect experiences in dreaming due to lack of temporal proximity to dreaming and specificity to a particular dream. We are currently working on a dream diary study that targets questions at a particular dream and is posed immediately after awakening which we consider a trustworthy methodology for eliciting conscious experience during sleep^90^. In addition, this approach allows for more open-responses to gain more granular detail.

A limitation of this study is its exploratory nature, which involved measuring multiple comparisons. While we adjusted for this using Benjamini and Hochberg's^52^ False Discovery rate, the findings should be interpreted as preliminary and suggestive of lines of inquiry for more direct experimental work. Our sample was also skewed towards zero scores on the CDS which means our analysis may miss nuance in understanding how our study variables were impacted across the range of low (rather than 0) to high DP scores. Future work in clinical samples would help unpack the relationship further.

Additionally, we partly used the online recruitment platform Prolific to recruit part of our sample, which may limit the generalizability of our findings. Future studies using more representative samples would be needed to confirm and extend our findings.

Finally, although the relationship between DP and anxiety is unclear^93^, future work in clinical samples may look to control for the mediating effects of psychopathologies like anxiety on the relationship between DP and the variables we used for this study.

Despite these limitations, our study helps to unpack the phenomenology of DP (in a non-clinical sample) in relation to the experience of the bodily self during waking and dreaming states.

Our findings provide some support for the continuity hypothesis of dreaming, which proposes that there is a continuity between waking and dreaming states, and that the content and experiences of dreams are influenced by the individual's waking experiences and psychological processes^91^. Our findings suggest that there is a relationship between waking anomalous bodily experience in DP and specific aspects of bodily and self experience in dreams including changes in bodily perception and presence. However, we are unable to make conclusions on whether there is continuity between specific aspects of bodily experience as the measures for waking DP and bodily experience in dreams assess different aspects of bodily experience. Using the same questionnaire to measure aspects of bodily self-consciousness for waking and dreaming experiences would enable more accurate insight into claims over continuity. Although additional research is needed, we can speculate that our initial findings of continuity between the experience of the bodily self during waking and dreaming states may indicate that the alterations in bodily self experience in DP is not caused by, or not only caused by, alterations in the brain’s processing of sensory signals because in dreams, most sensory experience (e.g. visual) is simulated, and not due to incoming sensory signals.

Interestingly, previous research has shown that increased lucidity during dreaming is associated with dreaming from a third-person perspective and the experience of dissociation, which may be linked to the feeling of a ‘secondary consciousness’ (a sense of background awareness)^92^. Given that we found a correlation between high CDS scores and increased outside observer experiences during dreaming, it would be interesting to investigate the extent of overlap between these experiences and lucid dreaming. Differences in phenomenal character between outside observer dreams in DP and lucid dreaming may prove fruitful in breaking down the nature of changes to spatiotemporal self-location during dreaming.

Our findings that individuals with high DP traits are more likely to report frequent nightmares suggest that the disturbing phenomenology of DP may impact the content of experience during dreaming as well as waking. Further work is needed to understand specific nightmare phenomenology in DP and how this relates to aspects of bodily self-consciousness.

Turning to our waking state findings, we found an association between high DP traits and an increased permeability of body boundaries. Given that the reduction of the salience of body boundaries is a relatively common experience in contemplative practices and that some practitioners can systematically bring on feelings of body dissolution^49,70^, further studies comparing meditative and DP experiences may build our understanding of the differences in valence and perceived control of this type of experience.

Finally, our findings provide further indirect evidence of alterations to interoceptive signal processing in individuals with high DP traits, and, when considered in context of existing literature, tentatively suggest that interventions focusing on building trust in bodily signals may be effective in reducing DP traits. A better understanding of the relationship between presence, bodily experience, and self, not just in waking, but across the sleep wake cycle can help identify underlying causes and guide potential diagnostic and therapeutic work.

### Supplementary Information

Supplementary Table 1.

## Data Availability

The datasets generated and/or analysed during the current study are available in the [OSF] repository, https://osf.io/7wv6j/?view_only=30d93cac439c475cbb98ad18c8408400.

## References

[1] Sierra M, David AS (2011). Depersonalization: A selective impairment of self-awareness. Conscious Cogn..

[2] Ciaunica A, Roepstorff A, Fotopoulou AK, Petreca B (2021). Whatever next and close to my self—the transparent senses and the “second skin”: Implications for the case of depersonalization. Front. Psychol..

[3] Ciaunica A, Seth A, Limanowski J, Hesp C, Friston KJ (2022). I overthink—Therefore I am not: An active inference account of altered sense of self and agency in depersonalisation disorder. Conscious. Cogn..

[4] Hunter ECM, Sierra M, David AS (2004). The epidemiology of depersonalisation and derealisation: A systematic review. Soc. Psychiatry Psychiatric Epidemiol..

[5] Sierra M, Berrios G (1998). E. Depersonalization: Neurobiological perspectives. Biol. Psychiatry.

[6] Sierra, M. in *Depersonalization: a new look at a neglected syndrome* (Cambridge University Press, 2009).

[7] Sierra M, David AS (2011). Depersonalization: A selective impairment of self-awareness. Conscious. Cogn..

[8] Medford N (2016). Emotional experience and awareness of self: Functional MRI studies of depersonalization disorder. Front. Psychol..

[9] Baker D (2003). Depersonalisation disorder: Clinical features of 204 cases. Br. J. Psychiatry.

[10] Michal M (2011). Base rates for depersonalization according to the 2-item version of the Cambridge Depersonalization Scale (CDS-2) and its associations with depression/anxiety in the general population. J. Affect. Disord..

[11] Qin P, Wang M, Northoff G (2020). Linking bodily, environmental and mental states in the self—A three-level model based on a meta-analysis. Neurosci. Biobehav. Rev..

[12] Gallagher S (2000). Philosophical conceptions of the self: Implications for cognitive science. Trends Cogn. Sci..

[13] Blanke O, Metzinger T (2009). Full-body illusions and minimal phenomenal selfhood. Trends Cogn. Sci..

[14] Merleau-Ponty M (1945). in Phénoménologie de la perception 13–14.

[15] James W (1890). in The principles of psychology.

[16] Varela FJ, Rosch E, Thompson E (1991). The Embodied Mind.

[17] Seth AK, Tsakiris M (2018). Being a beast machine: The somatic basis of selfhood. Trends Cogn. Sci..

[18] Park H, Blanke O (2019). Coupling inner and outer body for self-consciousness. Trends Cogn. Sci..

[19] Babo-Rebelo M, Richter CG, Tallon-Baudry C (2016). Neural responses to heartbeats in the default network encode the self in spontaneous thoughts. J. Neurosci..

[20] Legrand D (2006). The bodily self: The sensori-motor roots of pre-reflective self-consciousness. Phenomenol. Cogn. Sci..

[21] Simeon D (2000). Feeling unreal: A PET study of depersonalization disorder. Am. J. Psychiatry.

[22] Mantovani A (2011). Temporo-parietal junction stimulation in the treatment of depersonalization disorder. Psychiatry Res..

[23] Jay E, Sierra M, Van den Eynde F, Rothwell JC, David AS (2014). Testing a neurobiological model of depersonalization disorder using repetitive transcranial magnetic stimulation. Brain Stimul..

[24] Ionta S (2011). Multisensory mechanisms in temporo-parietal cortex support self-location and first-person perspective. Neuron.

[25] Perkins, J. in *Life on Autopilot: A Guide to Living with Depersonalization Disorder* (Jessica Kingsley Publishers, 2021).

[26] Ciaunica, A., Charlton, J. & Farmer, H. When the Window Cracks: Transparency and the Fractured Self in Depersonalisation. (2020).

[27] Mayer-Gross, W. On depersonalization. *Br. J. Med. Psychol*, 103–126 (1935).

[28] Revonsuo, A. in *Inner presence* (MIT Press., 2006).

[29] Revonsuo, A., Tuominen, J. & Valli, K. The Avatars in the Machine: Dreaming as a Simulation of Social Reality. *Open MIND* (2015).

[30] Windt JM (2015). in Dreaming.

[31] Windt JM (2018). Predictive brains, dreaming selves, sleeping bodies: How the analysis of dream movement can inform a theory of self- and world-simulation in dreams. Synthese.

[32] Schonhammer R (2005). 'Typical dreams' reflections of arousal. J. Conscious. Stud..

[33] Cheyne, J. A. Sleep paralysis and the structure of waking-nightmare hallucinations. *Dreaming (New York, N.Y.)* **13**, 163–179 (2003).

[34] Salami, A., Andreu-Perez, J. & Gillmeister, H. *Towards Decoding of Depersonalisation Disorder Using EEG: A Time Series Analysis Using CDTW*, IEEE, Piscataway, Dec 1, 2020).

[35] Sedeño L (2014). How do you feel when you can't feel your body? Interoception, functional connectivity and emotional processing in depersonalization-derealization disorder. PloS one.

[36] Schulz A (2015). Altered patterns of heartbeat-evoked potentials in depersonalization/derealization disorder: Neurophysiological evidence for impaired cortical representation of bodily signals. Psychosom. Med..

[37] Aspell JE (2013). Turning body and self inside out: Visualized heartbeats alter bodily self-consciousness and tactile perception. Psychol. Sci..

[38] Farmer H (2020). The detached self: Investigating the effect of depersonalisation on self-bias in the visual remapping of touch. Multisens. Res..

[39] Michal M (2014). Striking discrepancy of anomalous body experiences with normal interoceptive accuracy in depersonalization-derealization disorder. PloS one.

[40] Mehling WE, Acree M, Stewart A, Silas J, Jones A (2018). The Multidimensional assessment of interoceptive awareness, Version 2 (MAIA-2). PLoS ONE.

[41] Sierra M, Berrios GE (2000). The Cambridge Depersonalisation Scale: A new instrument for the measurement of depersonalisation. Psychiatry Res..

[42] Aponte-Soto MR, Vélez-Pastrana M, Martínez-Taboas A, González RA (2014). psychometric properties of the Cambridge depersonalization scale in Puerto Rico. J. Trauma Dissoc..

[43] Sugiura M (2009). Reliability and validity of a Japanese version of the Cambridge depersonalization scale as a screening instrument for depersonalization disorder. Psychiatry Clin. Neurosci..

[44] Migliorini V (2012). Italian (cross cultural) adaptation and validation of the Cambridge Depersonalization Scale (CDS). Epidemiol. Psychiatr. Sci..

[45] Sierra M, Baker D, Medford N, David AS (2005). Unpacking the depersonalization syndrome: An exploratory factor analysis on the Cambridge Depersonalization Scale. Psychol. Med..

[46] Noreika V (2020). Modulating dream experience: Noninvasive brain stimulation over the sensorimotor cortex reduces dream movement. Sci. Rep..

[47] Schredl M (2003). Effects of state and trait factors on nightmare frequency. Eur. Arch. Psychiatry Clin. Neurosci..

[48] Schredl M (2004). Reliability and stability of a dream recall frequency scale. Percept. Motor Skills.

[49] Dambrun M (2016). When the dissolution of perceived body boundaries elicits happiness: The effect of selflessness induced by a body scan meditation. Conscious. Cogn..

[50] Aron A, Aron EN, Smollan D (1992). Inclusion of other in the self scale and the structure of interpersonal closeness. J. Person. Soc. Psychol..

[51] Brown TA (2017). Psychometric evaluation and norms for the multidimensional assessment of interoceptive awareness (MAIA) in a clinical eating disorders sample. Eur. Eat. Disord. Rev..

[52] Benjamini, Y. & Hochberg, Y. Controlling the false discovery rate: A practical and powerful approach to multiple testing. *J. R. Stat. Soc. Ser. B Methodol.* **57**, 289–300 (1995).

[53] Windt JM (2010). The immersive spatiotemporal hallucination model of dreaming. Phenom. Cogn. Sci..

[54] Murray CD (2009). in Psychological Scientific Perspectives on Out of Body and Near Death Experiences.

[55] Lenggenhager B, Mouthon M, Blanke O (2009). Spatial aspects of bodily self-consciousness. Conscious. Cogn..

[56] Lenggenhager B, Tadi T, Metzinger T, Blanke O (2007). Video ergo sum: Manipulating bodily self-consciousness. Science.

[57] Hobson JA (1989). in Sleep.

[58] Hunt, H. T. in *Theœ multiplicity of dreams* (Yale Univ. Pr, New Haven u.a, 1989).

[59] Nielsen TA (2000). A review of mentation in REM and NREM sleep: “Covert” REM sleep as a possible reconciliation of two opposing models. Behav. Brain Sci..

[60] Bekrater-Bodmann R (2015). Post-amputation pain is associated with the recall of an impaired body representation in dreams—results from a nation-wide survey on limb amputees. PLoS ONE.

[61] Saurat M, Agbakou M, Attigui P, Golmard J, Arnulf I (2011). Walking dreams in congenital and acquired paraplegia. Conscious. Cogn..

[62] Voss, U., Tuin, I., Schermelleh-Engel, K. & Hobson, A. Waking and dreaming: Related but structurally independent. Dream reports of congenitally paraplegic and deaf-mute persons. *Conscious. Cogn.* **20**, 673–687 (2011). 10.1016/j.concog.2010.10.020 21147002

[63] Lusignan F (2009). Dream content in chronically-treated persons with schizophrenia. Schizophrenia Res..

[64] Hurovitz, C. S., Dunn, S., Domhoff, G. W. & Fiss, H. The dreams of blind men and women. *Dreaming (New York, N.Y.) 9*, 183–193 (1999).

[65] Rek S, Sheaves B, Freeman D (2017). Nightmares in the general population: Identifying potential causal factors. Soc. Psychiatry Psychiatr. Epidemiol..

[66] Wang J, Zemmelman SE, Hong D, Feng X, Shen H (2021). Does COVID-19 impact the frequency of threatening events in dreams? An exploration of pandemic dreaming in light of contemporary dream theories. Conscious. Cogn..

[67] Cheung, V. K. An exploratory study on the relationship between dissociation in waking life and negative contents in dreams (2012).

[68] Levin R, Nielsen TA (2007). Disturbed dreaming, posttraumatic stress disorder, and affect distress. Psychol. Bull..

[69] Agargun MY (2003). Nightmares and dissociative experiences: The key role of childhood traumatic events. Psychiatry Clin. Neurosci..

[70] Ataria Y, Dor-Ziderman Y, Berkovich-Ohana A (2015). How does it feel to lack a sense of boundaries? A case study of a long-term mindfulness meditator. Conscious. Cogn..

[71] Dor-Ziderman, Y., Ataria, Y., Fulder, S., Goldstein, A. & Berkovich-Ohana, A. Self-specific processing in the meditating brain: A MEG neurophenomenology study. *Neurosci. Conscious.* **2016**, niw019 (2016). 10.1093/nc/niw019 PMC6210398 30397512

[72] Lindahl JR, Britton WB (2019). 'I have this feeling of not really being here': Buddhist meditation and changes in sense of self. J. Conscious. Stud..

[73] Deane G, Miller M, Wilkinson S (2020). Losing ourselves: Active inference, depersonalization, and meditation. Front. Psychol..

[74] Millière R, Carhart-Harris RL, Roseman L, Trautwein F, Berkovich-Ohana A (2018). Psychedelics, meditation, and self-consciousness. Front. Psychol..

[75] Austin. Consciousness evolves when the self dissolves. *J. Consciousness Stud.* **7**, 209–230 (2000).

[76] Tsakiris M, Jiménez AT, Costantini M (2011). Just a heartbeat away from one's body: Interoceptive sensitivity predicts malleability of body-representations. Proc. R. Soc. B.

[77] Monti A, Porciello G, Panasiti MS, Aglioti SM (2022). The inside of me: Interoceptive constraints on the concept of self in neuroscience and clinical psychology. Psychol. Res..

[78] Friston K, FitzGerald T, Rigoli F, Schwartenbeck P, Pezzulo G (2017). Active inference: A process theory. Neural Comput..

[79] Gerrans P (2019). Depersonalization disorder, affective processing and predictive coding. Rev. Phil. Psych.

[80] Seth, A. K. & Critchley, H. D. An interoceptive predictive coding model of conscious presence. *Front. Psychol. *(2012). 10.3389/fpsyg.2011.00395 PMC3254200 22291673

[81] Sass LA (2014). Self-disturbance and schizophrenia: Structure, specificity, pathogenesis (Current issues, New directions). Schizophrenia Res..

[82] Sass LA, Parnas J (2003). Schizophrenia, consciousness, and the self. Schizophrenia Bull..

[83] Fuchs T (2015). Pathologies of intersubjectivity in autism and schizophrenia. J. Conscious. Stud..

[84] Craig AD (2002). How do you feel? Interoception: The sense of the physiological condition of the body. Nat. Rev. Neurosci..

[85] Seth AK (2013). Interoceptive inference, emotion, and the embodied self. Trends Cogn. Sci..

[86] Gerrans, P. Pain asymbolia as depersonalization for pain experience. An Interoceptive Active Inference Account. *Front. Psychol.* **11**, 523710 (2020). 10.3389/fpsyg.2020.523710 PMC7658103 33192765

[87] De Jaegher H, Di Paolo E (2007). Participatory sense-making. Phenomenol. Cogn. Sci..

[88] Sterling P (2012). Allostasis: A model of predictive regulation. Physiol. Behav..

[89] Ciaunica, A. & Fotopoulou, A. in *Embodiment, Enaction, and Culture* (The MIT Press, 2017).

[90] Windt JM (2013). Reporting dream experience: Why (not) to be skeptical about dream reports. Front. Hum. Neurosci..

[91] Hobson, J. A. in *Dreaming: An introduction to the science of sleep.* (Oxford Univ. Press, Oxford u.a., 2002).

[92] Voss U (2014). Induction of self awareness in dreams through frontal low current stimulation of gamma activity. Nat. Neurosci..

[93] Sierra M, Medford N, Wyatt G, David AS (2012). Depersonalization disorder and anxiety: A special relationship?. Psychiatr. Res..

[94] Simeon, D., & Abugel, J. *Feeling Unreal: Depersonalization Disorder and the Loss of the Self* (Oxford University Press, 2006).

[95] Sierra M (2009). Depersonalisation: A New Look at a Neglected Syndrome.

[96] Suksasilp C, Garfinkel SN (2022). Towards a comprehensive assessment of interoception in a multi-dimensional framework. Biol Psychol..

