# Supplementary material for: Examining the association between depersonalisation traits and the bodily self in waking and dreaming
Source: Sci Rep. 2024 Mar 13;14:6107. doi: 10.1038/s41598-024-56119-w (PMC10937666; doi:10.1038/s41598-024-56119-w)
Supplement: Supplementary file 1 — Supplementary Table 1. [file 41598_2024_56119_MOESM1_ESM.pdf]

Supplementary Table 1. Study hypotheses alongside correlations for full and modified samples.

Hypotheses		Full sample (n=514)	Removed 0 scores (total CDS) (n=502)	Included greater than or equal to 50 scores (total CDS) (n=223)
1a	Participants with higher DP traits (as measured by CDS-total score) will report that they trust internal bodily signals less while awake	r = -.52 (p<.01)	r = -.51 (p<.01)	r = -.34 (p<.01)
1b	Participants with higher DP traits will report that they notice internal bodily signals less while awake	r = -.08 (p>.05)	r = -.08 (p>.05)	r = -.03 (p>.05)
2a	Participants with higher DP traits will report that they have more dream experiences from an outside observer perspective	r = .28 (p<.01)	r = .29 (p<.01)	r = .26 (p<.01)
2b	Participants with higher DP traits will report that they are less aware of the presence (or absence) of their body in dreams.	r = .02 (p>.05)	r = .04 (p>.05)	r = -.02 (p>.05)
2c	Participants with higher DP traits will report that they have more dream experiences of distinct bodily sensations	r = .23 (p<.01)	r = .23 (p<.01)	r = .06 (p>.05)
2d	Participants with higher DP traits will report that they have more dream experiences of alterations in bodily perception	r = .24 (p<.01)	r = .25 (p<.01)	r = .16 (p<.01)
3	Participants with higher DP traits will report that the boundaries of their body are more permeable while awake	r = -.31 (p<.01)	r = -.32 (p<.01)	r = -.28 (p<.01)
4	Participants with higher DP traits will report that their sense of self is more separate from others while awake	r = -.01 (p>.05)	r = .01 (p>.05)	r = -.07 (p>.05)
5	Participants with higher DP traits will report more frequent nightmares	r = .33 (p<.01)	r = .32 (p<.01)	r = .17 (p<.05)
6	Participants with higher DP traits will report more frequent dream recall	r = .17 (p<.01)	r = .19 (p<.01)	r = .04 (p>.05)

Supplementary Table 1. Displays correlations for our study's hypotheses. Column 2 'Full sample (n=514)' shows the correlations for our full sample. Column 3 'Removed 0 scores (total CDS) (n=502)' shows the correlations for our sample with the removal of all participants who score for total CDS score (n=12). Column 4 'Included greater than or equal to 50 scores (total CDS) (n=223)' shows the correlations for our sample with the removal of all participants scoring under 50 total CDS score (n=291).
